# Development of a Coulometric Method for Assessing the Concentration of Ambient Levels of CO_2_/Air in Compressed Gas Mixtures

**DOI:** 10.6028/jres.096.031

**Published:** 1991

**Authors:** G. D. Mitchell, A. A. Bell

**Affiliations:** National Institute of Standards and Technology, Gaithersburg, MD 20899

**Keywords:** carbon dioxide, coulometry, current efficiency, cylinder gases, monoethanolamine, N,N-dimethylformatide

## Abstract

The coulometric method presented here is a reliable method for the direct analysis of CO_2_/air cylinder gas mixtures. It is based on Faraday’s laws of electrolysis and therefore no external standardization is required. A series of CO_2_/air cylinder gas mixtures ranging in concentration from 300 to 375 µmol/mol (ppm) were analyzed and the results compared to those results obtained by non-dispersive infrared (NDIR) analysis with traceability to gravimetric standards. The coulometric method is rapid, sensitive, precise, and with the proper experimental controls, will yield accurate results.

## 1. Introduction

The understanding of global “greenhouse” issues as they relate to CO_2_ in the atmosphere is a current environmental concern [1,2]. To assess changes in the levels of CO_2_ in the atmosphere, three considerations are important: 1) a substantial history of the global levels of CO_2_ over an extended period of time, 2) the fidelity of standards upon which the analyses are based, and 3) accuracy and reproducibility of methods used. At the National Institute of Standards and Technology there is a continuous search for methods of analysis that yield results that are traceable to fundamental quantities. The present method used for the certification of CO_2_/air cylinder gas mixtures as Standard Reference Materials is nondispersive infrared analysis which is calibrated with CO_2_ in air standards prepared by gravimetry [3]. In this work we present an analytical technique based on coulometry that provides accurate CO_2_ analysis for ambient-air standards having negligible quantities of other acid components. This technique can be used to assist in the validation of CO_2_/air SRMs.

In our companion paper [4] we described the design, construction, and use of an apparatus for the determination of SO_2_ in gas cylinder mixtures at the µmol/mol level. Here, the apparatus has been modified to demonstrate the potential utility of coulometry as an accurate and reliable method for assessing the concentration of CO_2_ at the 300 to 375 µmol/mol level in cylinder gas mixtures.

## 2. Experimental Methods

The apparatus and experimental details have been previously described [4] and will not be discussed fully here. However, because the analytical technique is being applied to CO_2_/air and not SO_2_/N_2_, the experimental differences will be delineated. The experimental method is based on acid-base coulometric titration with photometric end-point detection. The experimental objectives are to: (a) select a reaction scheme; (b) develop an accurate and precise sample delivery and absorption system; and (c) determine the current efficiency independently. The absorption system selected is designed to meet the two major criteria for coulometric titration, i.e., complete absorption of the CO_2_ analyte gas and near 100% current efficiency [5,6,7].

The absorbing solution consists of 78 mL N,N-dimethylformamide [HCON-(CH_2_)_2_], 2 mL 0.1% thymolphthalein (5′,5″-diisopropyl-2′,2″-dimethylphenolphthalein) dissolved in dimethylformamide, 3 g potassium iodide dissolved in 3 mL water, and 3 mL monoethanolamine (HOCH_2_CH_2_NH_2_). The solution is prepared in a 100 mL flask. The pH indicator, thymolphthalein, operates over the pH range from 9.3 to 10.5 and has an absorption maximum at 598 nm. All chemicals are reagent grade and are used without further purification. A possible reaction scheme involves the reduction of H_2_O at the cathode,

$$H_{2}O+e^{-}\to \frac{1}{2}H_{2}+{\text{OH}}^{-}$$ (1)

then

$$\begin{array}{l} K^{+}+{\text{OH}}^{-}+{\text{HOCH}}_{2}{\text{CH}}_{2}{\text{NH}}_{2}\to K^{+}\\ +{\;}^{-}{\text{OCH}}_{2}{\text{CH}}_{2}{\text{NH}}_{2}+H_{2}\text{O}\; \end{array}$$ (2)

Alternative schemes are presented in Ref. [5].

### 2.1 Procedure

The apparatus for CO_2_ analysis uses a narrow band interference filter (599 ± 10 nm) and a titration cell, filled with the absorbing solution described above. Light passing through the clear gas-absorbing solution causes the photodiode detector to generate a titration current that is fed back to the cathode, thus causing reaction [1] to occur. The pH indicator thymolphthalein is activated by the production of the base and the absorbing solution turns blue, which decreases the output of the photodiode. When the color of the absorbing solution reaches a selected level of intensity, the feedback circuit is adjusted to give a zero current output for the cell. The CO_2_/air mixture is introduced, metered by a mass flow controller calibrated to 0.1 mL/min. When the feedback circuit is adjusted to zero and the CO_2_/air mixture is introduced into the cell, the analytical procedure and data analysis are identical to those discussed in Ref. [4].

The critical measurement parameters are cell current (A) and flow rate (mL/min). The current is measured using a calibrated digital current meter and the flow rate is monitored using a calibrated mass flow controller. The relationship of cell current and flow rate of CO_2_ is linear over the experimental range. Equation (3) gives the relationship of cell current and flow rate as it is used to determine CO_2_ concentration in µmol/mol.

$${\text{CO}}_{2}\left(\frac{\mu {\text{mol}\;\text{CO}}_{2}}{\text{mol}\;\text{air}}\right)=\frac{C_{\text{cell}}\cdot K\prime }{Q}$$ (3)

where

*C*_cell_ = Cell current (A)
*Q* = Flow rate of sample (mL/min)

and

$$K\prime =\frac{6\cdot 10^{7}\cdot MV_{T}}{n\cdot F}$$ (4)

*MV_T_* = Molar volume of air (corrected for temperature) mL/mol (*MV_T_* = 24475.59 @ 298 K)
*n* = Number of electron change (e^−^) (*n* = 1)
*F* =The Faraday constant (96485.38 A s/mol)
6·10^7^ is a unit conversion factor

$$\left(\frac{s\cdot \mu \text{mol}}{\text{min}\cdot \text{mol}}\right)$$

## 3. Results and Discussion

**Figure 1:**
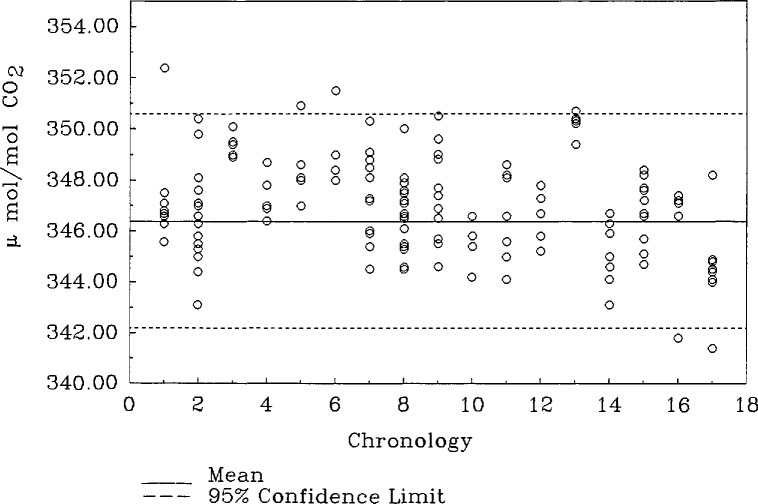
Chronological study.

Ten compressed gas cylinders with CO_2_ concentrations from 300 to 375 µmol/mol were previously analyzed by a non-dispersive infrared (NDIR) analyzer calibrated with gravimetrically prepared standards [3]. A cylinder representing the mid-range of the set of 10 cylinders was selected as a control (cylinder 4, Table 1) to be used throughout this study to evaluate the overall uncertainty associated with the coulometric method and the day to day imprecision of the analysis. The control was studied over a variety of experimental conditions and for an extended period of time. The control cylinder was analyzed by the coulometric method on 17 separate occasions. Figure 1 shows the uncorrected data for the control cylinder as they were obtained chronologically over a 11 month period. The mean and the 95% confidence interval are indicated by the horizontal solid and dashed lines, respectively. The best estimate of the true mean concentration and its uncertainty at the 95% level of confidence is 346.4 ± 4.2 µmol/mol. For any given day, the standard deviation of a single measurement for the concentration range from 300 to 375 µmol/mol CO_2_/air is 2.1 µmol/mol. The reported NDIR concentration of the control cylinder was 341.7 ± 0.4 µmol/mol. The comparison of this value to the coulometrically determined value is a measure of the current efficiency. At the 95% level of confidence, the current efficiency is calculated to be 101.3 ± 1.0%. This value represents a systematic bias in the measurement system and gives the factor by which the experimental data should be corrected.

Results for the coulometric analysis of the 10 CO_2_/air gas cylinders mixtures as well as those obtained by NDIR are given in Table 1. The difference between the NDIR and corrected coulometric analytical values range from 0.003 to 1.1%. These results, however, show that at the present stage of development the method is capable of producing results that are comparable to those of the reference method. Figure 1 shows that the resulting analysis of the control cylinder is biased by 1.3%. No explanation for the bias is being offered at the present, however, future work is planned to improve both the precision and accuracy of the method.

## 4. Conclusion

A coulometric method for the direct analyses of reference cylinder gas mixtures has been demonstrated. It has been shown to be sensitive, potentially precise and accurate for determining CO_2_ in air at atmospheric concentrations, as well as a viable method of assessing the concentration of CO_2_ cylinder gas mixtures. The system presented here provides rapid analyses, and is capable of being independent in the determination of CO_2_ at ambient air levels. The upper and lower limits of detection have not yet been fully explored. However, on the upper end, the limiting critical component of this approach is the ability of the absorbing solution to handle higher concentrations of CO_2_. On the low end, preliminary tests have provided detection limits of approximately 1 µmol/mol CO_2_/air. This method of analysis is traceable to the Faraday constant and therefore no standardization against analyzed samples or pure materials is required. At the present state of development, the imprecision of a single analysis by the coulometric method is approximately 1.3%.

## Figures and Tables

**Table 1:** Comparison of CO_2_ coulometric^a^ and NDIR data

Cylinder number	Direct coloumetric concentration µmol/mol	Corrected^b^ concentrations µmol/mol	NDIR concentration^c^ µmol/mol	Ratio Coul/NDIR
1	307.3	303.3	303.8	0.998
2	337.5	333.1	335.9	0.992
3	338.1	333.7	335.9	0.993
4^*^	346.4	341.9	341.7	1.001
5	344.8	340.3	342.8	0.993
6	348.2	343.7	343.2	1.001
7	349.8	345.2	347.6	0.993
8	352.7	348.1	351.5	0.990
9	380.2	375.3	375.2	1.000
10	380.7	375.8	375.3	1.001

a Uncertainty ± 4.2 µmol/mol.

b Direct coulometric data corrected for current efficiency. $\text{Current}\;\text{efficiency}=\frac{\text{Coulometric}\;\text{current}\;\left(\text{mA}\right)}{\text{Theoretical}\;\text{current}\;\left(\text{mA}\right)}\times 100$.

c Uncertainty ±0.4 µmol/mol.
